# Micronutrients in HIV: A Bayesian Meta-Analysis

**DOI:** 10.1371/journal.pone.0120113

**Published:** 2015-04-01

**Authors:** George M. Carter, Debbie Indyk, Matthew Johnson, Michael Andreae, Kathryn Suslov, Sudharani Busani, Aryan Esmaeili, Henry S. Sacks

**Affiliations:** 1 Foundation for Integrative AIDS Research, Brooklyn, NY, United States of America; 2 Icahn School of Medicine at Mount Sinai, New York, NY, United States of America; 3 Teachers College, Columbia University, New York, NY, United States of America; 4 Department of Anesthesiology, Albert Einstein College of Medicine, Bronx, NY, United States of America; China Medical University, CHINA

## Abstract

**Background:**

Approximately 28.5 million people living with HIV are eligible for treatment (CD4<500), but currently have no access to antiretroviral therapy. Reduced serum level of micronutrients is common in HIV disease. Micronutrient supplementation (MNS) may mitigate disease progression and mortality.

**Objectives:**

We synthesized evidence on the effect of micronutrient supplementation on mortality and rate of disease progression in HIV disease.

**Methods:**

We searched MEDLINE, EMBASE, the Cochrane Central, AMED and CINAHL databases through December 2014, without language restriction, for studies of greater than 3 micronutrients versus any or no comparator. We built a hierarchical Bayesian random effects model to synthesize results. Inferences are based on the posterior distribution of the population effects; posterior distributions were approximated by Markov chain Monte Carlo in OpenBugs.

**Principal Findings:**

From 2166 initial references, we selected 49 studies for full review and identified eight reporting on disease progression and/or mortality. Bayesian synthesis of data from 2,249 adults in three studies estimated the relative risk of disease progression in subjects on MNS vs. control as 0.62 (95% credible interval, 0.37, 0.96). Median number needed to treat is 8.4 (4.8, 29.9) and the Bayes Factor 53.4. Based on data reporting on 4,095 adults reporting mortality in 7 randomized controlled studies, the RR was 0.84 (0.38, 1.85), NNT is 25 (4.3, ∞).

**Conclusions:**

MNS significantly and substantially slows disease progression in HIV+ adults not on ARV, and possibly reduces mortality. Micronutrient supplements are effective in reducing progression with a posterior probability of 97.9%. Considering MNS low cost and lack of adverse effects, MNS should be standard of care for HIV+ adults not yet on ARV.

## Introduction

Antiretroviral therapy (ARV) has revolutionized care and outcomes for HIV infected individuals. However, it remains out of reach for many, due to cost and other barriers.[1] UNAIDS/WHO estimated the number of people living with HIV in 2012 to be 35.3 million (range 32,200,000–38,800,000)[2], some 25 million of whom live in sub-Saharan Africa (23.5–26.6 million). While globally 12.9 million individuals are receiving ARV, over 25 million people who are currently clinically eligible (CD4<500) to receive ARV do not yet have access.[3] Despite increasing availability of ARV, in 2013 alone, some 1.5 million men, women and children died of AIDS.[4]

Vitamins and certain minerals (micronutrients) are essential for metabolic and immunologic functions. Micronutrient (MN) deficiencies arise due in part to inflammatory and immunological effects of HIV infection in the gut, which impairs absorption. In addition concurrent infections, antibiotic and other drug treatment may contribute to deficiencies.[5] Multiple studies have documented declines in peripheral blood micronutrient status, some showing greater declines in fat-soluble vitamins and selenium at all disease stages compared to water-soluble vitamins and some minerals.[6,7] “Multiple” supplements are routinely available over-the-counter and widely used by people with HIV disease.[8]

A prior meta-analysis was unable to make conclusive findings with regard to an effect on disease progression rate or mortality.[9] Bayesian techniques permit synthesis of more sources of evidence (i.e., randomized controlled trials (RCTs), observational studies, expert judgment) within a single coherent model.[10] Bayesian analysis provides a robust credible interval of probabilities that the treatment is effective even with small numbers of included participants or trials, where frequentist confidence intervals often lack correct coverage probabilities for small sample sizes. Our analysis incorporates evidence from a recent large trial,[11] permitting us to synthesize data and provide more definitive results, even in the context of estimated high between study variability.

### Objective

Our objective was to synthesize the evidence of the effects of multiple micronutrient supplementation (MNS) reporting on the effects of MNS on mortality and the rate of disease progression among HIV+ adults not on ARV.

## Methods

Our protocol was registered with PROSPERO[12] and we followed the PRISMA Guidelines in the preparation of this manuscript.[13]

### Eligibility Criteria

We included clinical trials of individuals of any age with documented HIV infection with or without concurrent or active opportunistic or other infection and not on antiretroviral therapy. Most over-the-counter MNS contain more than three ingredients. We included studies that investigated more than 3 micronutrients, as in other studies,[9] versus any or no comparator and reported disease progression or mortality as outcomes, imposing no language or time restrictions. We defined micronutrients as supplemental vitamins and/or additional minerals as a multivitamin (MV), multivitamin with minerals (MVM) or other combination of micronutrient supplements (MNS) in tablet, powder, capsule, softgel or fortified food forms.

### Information Sources

We searched MEDLINE, EMBASE, the Cochrane Central Register of Controlled Trials (The Cochrane Library), AMED and CINAHL.

### Search

Databases were queried with the same search terms, tailored to the particular database. Our search terms are described in the Prospero protocol. Searches were conducted from database inception through December 2014. Review articles were assessed for additional references.

### Study Selection

Two reviewers independently screened citations and abstracts of all publications obtained by the search strategies. Disagreements were resolved by consensus.

### Data Collection Process

For eligible trials, we obtained full articles and assessed their relevance based on the preplanned criteria for inclusion in the systematic review.

### Data Items

We extracted data on study design, intervention, site, number of participants, inclusion and exclusion criteria, duration, toxicity/adverse events, primary endpoint, secondary endpoint, findings, baseline differences, conclusions, efficacy and safety.

### Risk of Bias of Individual Studies

We evaluated methodological quality based on the Jadad score,[14] assessing sequence generation, allocation concealment, blinding, intention to treat or per protocol analysis, missing data and attrition, and selective reporting. We followed the MOOSE Guidelines for Meta-Analyses and Systematic Reviews of Observational Studies in our assessment of observational studies[15] as well as the guideline of the Newcastle-Ottawa scale.[16]

### Summary Measures: Progression

We built a hierarchical Bayesian random effects model to synthesize results. Inferences are based on the posterior distribution of the population effects; posterior distributions were approximated by Markov chain Monte Carlo in OpenBugs.[17]

The hierarchical Bayesian model for the progression data synthesizes the reported hazard rates from the studies, and their associated standard errors. Because we did not have sufficient information to develop a model of the standard errors, we assume they represent the true variation of the estimated relative risk around its true value. That is, we assume

$$\log\left(HR_{ij}\right)∼N\left(\theta_{ij},s_{ij}^{2}\right),$$

where *HR*_*ij*_ is the estimated hazard rate in the *j*th arm of study *i*, *s*_*ij*_ is the reported standard error of the log-hazard rate in arm *j* of study *i*, and *θ*_*ij*_ is the true hazard rate of arm *j* in study *i*. The hierarchical random effects model further assumes that each true log hazard rate is normally distributed around a study mean log-hazard rate, *v*_*i*_, with variance *ω*_i_, i.e.,

$$\theta_{ij}∼N\left(\nu_{i},\;\omega_{i}\right),$$

with study mean log-hazard rates assumed to be normally distributed around a population mean log-hazard rate *μ* with variance equal to *τ*,

$$\nu_{i}∼N\left(\mu ,\tau \right).$$

A non-informative normal prior distribution with mean zero and variance equal to 10^5^ is assumed for the population parameter *μ*, and folded Cauchy distributions with scale parameters of 0.1 are utilized for the variance component *ω* and *τ*.

### Summary Measures: Mortality

The likelihood for our Bayesian hierarchical model of the mortality data assumes a probit regression model, where the observed fatalities in each arm of each study are assumed to be binomial random variables. The rate of fatalities in the control arm of study *i* is defined by an intercept *v*_*i*_ and the mortality rates in the (possibly multiple) treatment arms are assumed to be defined by the sum of the intercept *v*_*i*_, a study-level treatment effect *δ*_*i*_, and a arm-specific treatment effect *γ*_*j(k)*_. That is, the mortality rates for treatment condition *k* nested in arm *j* (*j = 0* for control and 1 for treatment) in study *i* is defined by the probit regression model,

$$p_{ij(k)}\;=\;{\Phi (\nu }_{i}+j(\delta_{i}+\gamma_{j\left(k\right)})),$$

where *Φ* is the cumulative distribution function of the standard normal distribution. The model further assumes that the intercepts and treatment effects are normally distributed random effects centered at population effects *v* and *δ*.

$$\nu_{i}∼N\left(\nu ,\;\tau_{\nu }\right)\;\mathrm{and}\;\delta_{i}∼N\left(\delta ,\;\tau_{\delta }\right)$$

and that the arm-specific treatment effects are normally distributed with a mean of zero and variance *τ*_*γ*_. The population mortality rates under control and treatment are defined as the probability that a randomly selected subject in a randomly selected study does not survive past the duration of the study,

$$p_{0}=\;\Phi \left(\frac{\nu }{1+\tau_{\nu }}\right)\;\mathrm{and}\;p_{1}=\;\Phi \left(\frac{\nu +\delta }{1+\tau_{\nu }+\tau_{\delta }+\tau_{\gamma }}\right).$$

The prior distribution for the population effects *v* and δ are non-informative normal distributions centered at zero with variances equal to 10^5^. The prior distributions of the variance parameters *τ*_*v*_, *τ*_*δ*_, and *τ*_*γ*_ are all folded Cauchy distributions with scale parameter equal to 0.1.

### Number Needed to Treat

The number needed to treat (NNT) is defined as $\frac{1}{p_{0}-p_{1}},$ where *p*_*0*_ and *p*_1_ are the probability of events (e.g., progression) under the placebo and MNS conditions. In the case of mortality data we approximate the posterior distribution of the NNT using the population proportions defined in the model description above. For disease progression, our model only utilized study information about relative risks and therefore we only have information about the ratio $\frac{p_{1}}{p_{0}}$. To estimate a number needed to treat for progression we used a 30% baseline progression rate (i.e., $p_{0}=0.3)$, which is similar to the progression rate of the placebo arm of the Fawzi [18] study, which had a median duration of follow-up of 60 months.

### Synthesis of Results

We undertook an assessment of heterogeneity and calculated a Bayes Factor for the likelihood of a 0 value for between-study heterogeneity.[19] In sensitivity analyses, we conducted a frequentist meta-analysis of the random effects for risk ratios (S1 and S2 Figs.).

### Risk of Bias Across Studies

We searched for evidence of selective reporting and for conflicts of interests, including eliciting the funding source of included studies. We attempted to assess publication bias.[20]

### Additional Analyses

We reviewed studies for adverse events and summarized those studies excluded from our formal meta-analysis. Frequentist analyses were conducted for sensitivity (S1–S4 Figs.). We undertook a meta-analysis using a random effects method for risk ratios. For each of the studies we input the reported log risk ratio and the reported standard error of the log risk ratio. The method explicitly avoids double-counting placebo recipients.

## Results

### Study Selection

**Fig 1:**
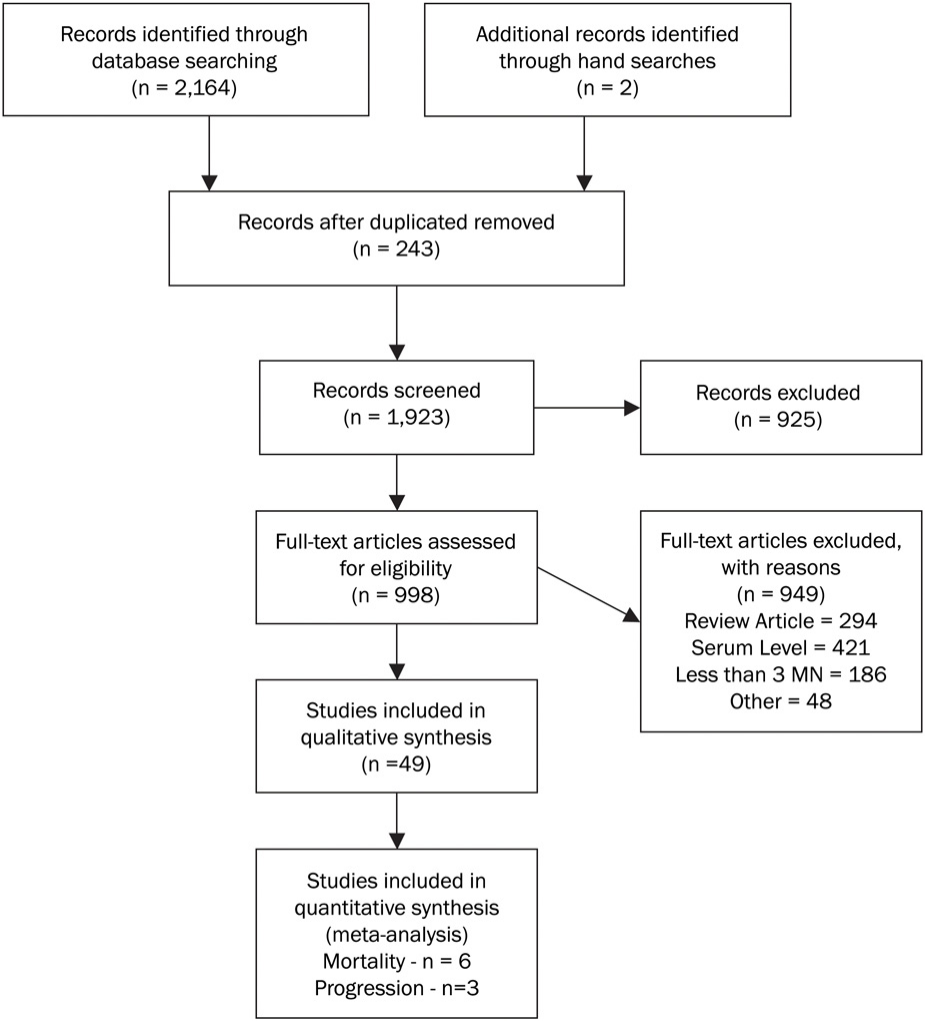
Flow Chart of Selection Process of Reviewed Studies.

Two reviewers extracted data from 49 of the initial 2,166 studies identified (Fig. 1). Of these 49, 8 reported pertinent data, 7 for mortality and 3 for progression.[18],[21],[22],[11],[23],[24],[25],[26],[27] The Baum[11] and Fawzi[18] studies reported on both outcomes (Table 1). Inquiries with non-medical practitioners or providers, patients or community groups outside the medical establishment or other grey literature yielded no data.[28]

**Table 1:** Summary of Data Extraction.

Reference	Location	Duration	Participants	Intervention	Control	Design	Jadad	Conclusions
Abrams, 8100273; 1993	San Francisco, CA	72 months	San Francisco Men’s Health Study n = 296 HIV+ 25–50 yo gay men not on ARV	Each participant asked to record brand, frequency and amount of each supplement taken	None	Prospective, observational study	NA	High nutrient intake at baseline in HIV+ men was assoc. w/higher CD4 count and a reduced risk of AIDS; hazard ratio (HR) = 0.7; 95% CI = 0.5, 1.0
Baum, 24281460; 2013	Botswana	24 months	N = 875 HIV+ adults not on ARV, CD4>350	B1-20 mg; B2-20 mg; B6-25 mg; Niacin-100 mg; B12-50 μg; C-500 mg; E-30 mg; Folic acid-0.8 mg; Se-200 μg	Placebo	RCT, factorial design	5	Long term MVI was safe and significantly prolonged time to CD4 <250; HR = 0.46, 95%CI: 0.25, 0.85, p = 0.01; *MV alone*: 0.54 (0.30, 0.98) p = 0.04; *MV+Se*: 0.48 (0.26, 0.88) p = 0.02; **#Deaths:** *Placebo*-1; *MNS+Se*-1
Fawzi, 15229304; 2004	Tanzania	71 months	n = 1,078 pregnant women; placebo-267; MV only-271; MV+A -268; A only-272	**Arm 1:** Vitamin A (5000 IU A Plus Beta carotene-30 mg.; **Arm 2**: Multi, no A; B1-20 mg; B2-20 mg; B6-25 mg; Niacin-100 mg; B12-60 μg; C-500 mg; E-30 mg; Folic acid-0.8 mg; **Arm 3:** MV+A	Placebo	RCT, 2x2 factorial design; Placebo vs. Vit A alone vs. MVs w/o Vit A	5	Progression reduced to stage 4; higher CD4 and lower viral load in MVI arm; RR 0.50 (0.28–0.90) p<0.02 over mean 61.4 months; *MV alone*: 0.50 (0.28, 0.90) p = 0.02; *MV+extra Vitamin A*: 0.67 (0.39, 1.15) p = 0.15; **#Deaths** (from AIDS-related causes): *Placebo*: 66/267; *MN*: 52/271 (p = 0.09); *MN + Vit A*: 60/268 (p = 0.58)
Jiamton, 14600517; 2003	Thailand	11 months	HIV+ adults with CD4 of 50–550; n = 481	A-3000 μg; Beta-carotene-6 mg; D3-20 μg; E-80 mg; K-80 μg; C-400 mg; B1-24 mg; B2-15 mg; B6-40 mg; B12-30 μg; Folacin-100 μg; Pantothenic acid-40 mg; Fe-10 mg; Mg-200 mg; Mn-8 mg; Zn-30 mg; I-300 μg; Cu-3 mg; Se-400 μg; Cr-150 μg; Cystine-66 mg	Placebo	RCT	5	**#Deaths**: *Placebo*: 15/239; *MN*: 8/242; **Mortality Hazard Ratios (MHR)**: Among pts with CD4 <200, MHR = 0.37 (0.13–1.06, p = 0.05); Among pts with CD4< 100: MHR =; 0.26 (0.07–0.97, p = 0.03)
Kelly, 18842788; 2008	Zambia	40 months	HIV-/HIV+ adults: n = 500 (total); n = 148 (HIV+)	A-10,500 IU; C-300 mg; E-300 mg; Se-150 μg; Zn-200 mg	Placebo	RCT, cluster randomized; midpoint crossover	5	**#Deaths** in first 12 mo. of study (among HIV+ participants): *Placebo*: 6/79; *MN*: 3/69; **#Deaths** in 36 mo. (among HIV+ participants): *Placebo*: 12/79–11/79 according to supplemental table; *MN*: 4/69
Range, 16571156; 2006	Tanzania	8 months	Adults w/ TB, mixed HIV status: n = 499 (total); n = 213 (HIV+)	**Zinc**-45mg; *or* **MNS**: A-5000 IU; B1-20 mg; B2-20 mg; B6-25 mg; B12-50 μg; Folic acid-0.8 mg; Niacin-40 mg; C-200 mg; E-60 mg; D3-200 IU; Se-0.2 mg; Cu-5 mg	Placebo	RCT, 2x2; MNS +placebo vs. MNS+Zn vs. Zn+plac. vs. placebo/placebo	5	**#Deaths** (among HIV+ participants): *Placebo only*: 14/42 (33.3% mortality rate); *Placebo+Zn*: 13/52 (25.0%); *MVM*: 11/45 (24.4%); *MVM+Zn*: 4/42 (9.5%)
Semba, 17705950; 2007	Malawi	24 months	Adults w/ TB, mixed HIV status: n = 1402 (total); n = 829 (HIV+)	A 8000 IU; C-500 mg; D-400 IU; E-200 IU; B6-2 mg; B12-6 μg; B1-1.5 mg; B2-1.7 mg; Niacin-20 mg; Folate-0.4 mg; Zn-10 mg; I-175 μg; Se-65 μg	Placebo	RCT	5	**#Deaths** (among HIV+ participants): *Placebo*: 171/423; *MN*: 157/406
Villamor, 18471061; 2008	Tanzania	43 months	Adults with TB: n = 887 (total); n = 471 (HIV+)	Retinol-5000 IU; B1-20 mg; B2-20 mg; B6-25 mg; B12-50 μg; Niacin-100 mg; Folic acid-0.8 mg; C-500 mg; E-500 mg; Se-100μg	Placebo	RCT	5	**#Deaths** (among HIV+ participants): *Placebo*: 66/238; *MN*: 74/232

### Risk of Bias Within Studies

The Jadad scale (a score ranging from 1 to 5) was used to assess bias within studies.[14] Each of the other studies was rated 5, indicating overall high methodological quality, with adequate well-described randomized allocation, allocation concealment and participants and observer blinding (Table 1). One included study was a prospective observational study,[21] which rated 5/9 on the Newcastle-Ottawa scale (S1 Table).[16] An analysis of publication bias by using a funnel plot or the test proposed by Egger was precluded by the small numbers of studies.[20] We found no evidence of selective reporting or conflicts of interests, with all studies funded by public entities.

### Synthesis of Results

#### Micronutrient supplementation slows progression to clinical disease/AIDS

**Fig 2:**
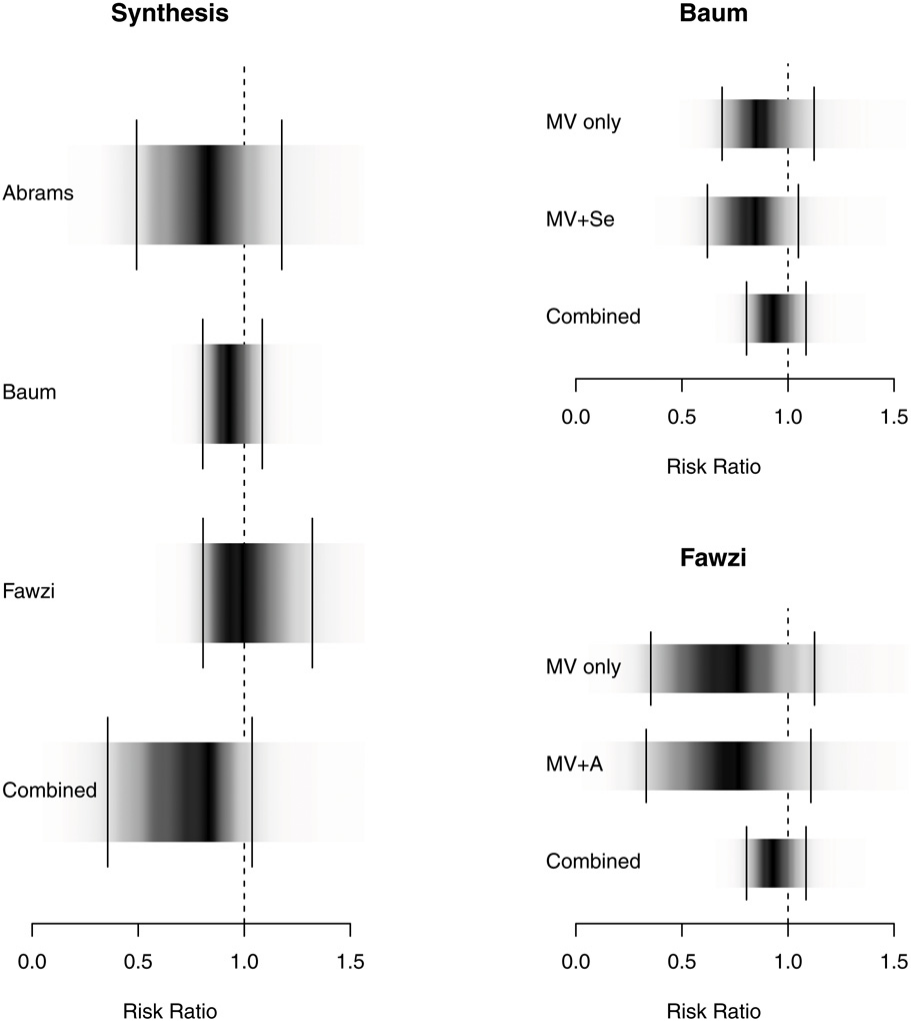
Density Strip Plot of Relative Risk of Progression to Clinical Disease/AIDS. Bayesian random effects analysis (favors treatment on the left of 1.0); density plots on the figure on the left represent combined data; on the right accounts for individual study arms and the impact of adding selenium or vitamin A to MNS [55].

Synthesis of data from 2,249 participants in 3 trials shows that MNS reduces the risk of disease progression.[11],[21],[18] The mean RR in our Bayesian model is 0.62 (credible interval, 0.37, 0.96). The posterior probability that micronutrient supplementation is effective in reducing the rate of progression is 98.2%, with a number needed to treat (NNT) of 9.7 (4.8, 28.9). The Bayes Factor is 53.4 indicating strong evidence for effectiveness[19] (Table 1 and Fig. 2). We used a conservative, two-tailed scale parameter representing a presumption of high between study variability, to reflect the fact that the individual studies were in different settings (industrialized versus developing countries).

#### Sensitivity analysis

When we used a very flat, uninformative and highly conservative parameter modeling a large between study variability with OR differences between studies of around 3[29] and prior to the Baum study data[11], we found the credible interval crossed 1, though 95.5% of the probability lies below 1 (RR 0.66; credible interval 0.35, 1.18) with a Bayes Factor of 21.2, still indicating strong evidence for effectiveness.[19]

Further, a sensitivity analysis employing frequentist methodology yielded a similar 40% reduction in the rate of progression to clinical disease stages (RR = 0.60; 0.46, 0.78; p = 0.00008) for subjects on MNS (S1 Fig.). Omitting the observational study slightly improved the point estimate and narrowed the credible interval.

#### Mortality

**Fig 3:**
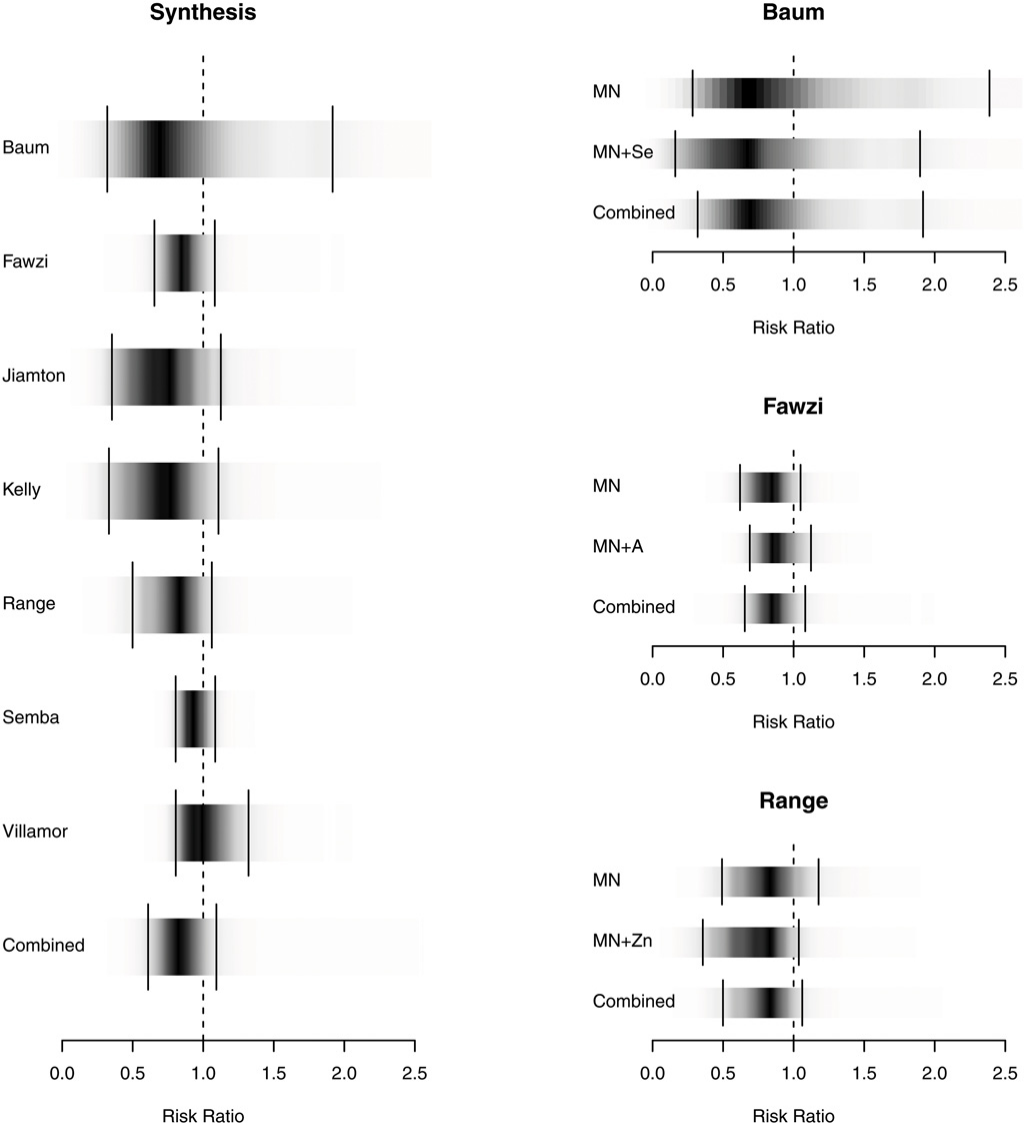
Density Strip Plot of Effect of MNS on Mortality. Bayesian random effects analysis (favors treatment on the left of 1.0); density plots on the figure on the left represent combined data; on the right accounts for individual study arms and the impact of adding selenium, vitamin A or zinc to MNS.

We identified seven pertinent RCTs with data (see Table 1) from 4,095 adults, the RR for mortality was 0.84 (95% credible interval, 0.38–1.85) (Fig. 3). Of note, in 3 of the studies, subjects were co-infected with tuberculosis.[25],[27,30] This represented 1,513 subjects. We estimated the NNT as 25 to prevent one death, with a credible interval of (4.3, ∞), reflecting our inability to conclude statistically that micronutrients reduce mortality rates. However, the posterior probability that micronutrients reduce mortality is 91.4%. The Bayes Factor (BF), a measure of the increased credibility of the hypothesis based on the new data was 10.6, suggesting positive evidence for the effectiveness of micronutrients according to the guidelines of Kass & Raftery.[19] Notably, one study observed a mortality benefit among those with CD4<100 (p = 0.03)[23] underscoring the importance of baseline CD4 count along with study duration. In a sensitivity analysis using frequentist techniques, mortality across studies yielded a RR of 0.85 (0.70, 1.05; p = 0.12) (S2 Fig.).

We performed a study-level sensitivity stratified analysis controlling for TB co-infection. In a frequentist analysis, we estimated a RR of 0.70 (0.53, 0.93; p = 0.02) for the studies not including patients with TB (S3 Fig.). For those studies including patients with TB at baseline, the RR is 0.91 (0.64, 1.31; p = 0.62) (S4 Fig.). This frequentist analysis estimates the between-study variability as zero between the non-TB studies (*τ* ^*2*^ *= 0*) (S3 Fig.).

Our Bayesian analysis, which assumed a non-zero between study variability, found that the posterior probability that micronutrients reduce mortality rates in the non-TB population is 96%; the estimate of the risk ratio is 0.73 (0.47, 1.10; p = 0.04). The estimate for the studies including patients with TB at baseline is RR 0.93 (0.59, 1.32; p = 0.29). The Bayesian approach led to wider credible intervals, because of the assumption of a non-zero between study variability.

#### Adverse events

6 of 8 studies explicitly reported on adverse events. Of these, none reported any serious side effects from MNS, with minor events being reported in one study more frequently among placebo recipients.[23] Assessment of the 49 studies qualitatively evaluated revealed no evidence of adverse events. Of note, however, Vitamin A/beta carotene and selenium were suggested to increase vaginal shedding of HIV.[31],[32]

## Discussion

### Summary of Evidence

Our evidence synthesis demonstrates that micronutrient supplementation substantially and significantly reduces the risk of HIV disease progression by 38% in adults not on ARV therapy. For disease progression, the NNT is 9.7. The probability that MNS slow HIV disease progression is 98.2%. The slowed rate of progression offers individuals not yet on ARV a potentially life-extending stopgap as the goal of ARV access for all remains elusive. Other clinical benefits may additionally accrue (discussed below), with little evidence of risk of harm or excessive cost.

For mortality, the NNT was 25 (credible interval, 4.3—∞), with a 91% probability that MN supplement reduces mortality. This is particularly evident among individuals with low CD4 counts, although clearly such individuals should be on ARV therapy. With the current dataset, we cannot definitively assert an effect of MNS on mortality.

In our stratified analysis controlling for opportunistic co-infection, we found a stronger effect in studies that did not include patients with TB at baseline, both in the frequentist and the Bayesian analysis. We hypothesize that micronutrients may moderately reduce mortality risk among adults without concurrent opportunistic infection. However, we caution that this was an *ex post facto* exploratory analysis.

Our Bayesian analysis included a positive value for between study variability, estimated to be 0.02 (0.0006, 0.15), i.e., about 2% of the observed variation could be said to be between study variation.

#### Clinical impact and implications

Our analyses demonstrate a significant and marked clinical benefit for adults with HIV not yet on ARV. However, benefits of MNS for those not on ARV should not serve as a rationale for delaying[33] or deferring[34] access to ARV.

The annual cost per patient of MNS reported ranged from about $12 to $40/patient/year. One of the least costly ($12/pt/year) yet most comprehensive MVM formulae was the one clinically evaluated by Jiamton’s group.[35] Fawzi’s group estimated a cost of $15/pt/year.[18] A global perspective on managing malnutrition among women and children has provided a figure of $9.6 billion to achieve 90% coverage in 34 focus countries.[36] A formal cost-effectiveness analysis would be useful; however, because of the very low cost of MNS, it is likely to be favorable.

Future studies might assess optimized formulae and dosages. In the Fawzi study, increasing the dosage of the vitamin A may have blunted the impact on progression rate.[18] By contrast, Baum’s group found that adding the mineral selenium improved the outcome significantly when adjustment was made for the interaction (0.46, 95% CI 0.25, 0.85).[11] The addition of zinc to a MNS reduced mortality (RR 0.29; 95% CI 0.10, 0.80).[25] Range et al. reported that zinc supplementation increased CD4 counts.[37] Vitamin D3 was not included in some formulae, while a variety of studies have underscored widespread deficiency among HIV+ individuals and a relationship between deficiency and poor outcomes.[38,39]

Others have noted numerous clinical benefits of MNS were reported, including improved birth outcomes and gastrointestinal function, reduction in hospital stay, improvement in hematology and quality of life.[40,41] Other data suggest on the one hand an increased resistance to malaria but potential for increase parasitemia of uncertain clinical significance when a MNS is provided [42].

MNS formulae that include a comprehensive array of vitamins and minerals (such as potassium, calcium, magnesium and selenium) may be more biologically relevant in the context of gut damage, mitochondrial toxicity, increased resting energy expenditure and oxidative stress observed in HIV infection and with chronic ARV treatment.[43] The finding that pyroptosis is a primary feature of HIV pathogenesis through the inflammatory suicide of bystander CD4 cells[44] generates a hypothesis of the potential benefit of limiting this effect via MNS.[45]

The strongest effect on mortality among those with CD4<200 used a formula with a range of micronutrients including vitamin D3, zinc, copper and selenium, which also happened to be one of the least expensive.[23] However, studies of the effect of MNS on mortality for people on ARV would be prohibitively large and long to ascertain any effect.

Use of MN supplements in the context of ARV therapy should be further assessed. While one study using a comprehensive intervention with high dosages showed increased CD4 among North Americans on ARV [43], another among Tanzanians was stopped early due to elevations in ALT liver enzymes [46]. Research might also be directed to assess the potential for drug interactions, side effects (such as increased ALT among those on ARV) or ancillary benefits such as reducing or limiting neuropathy[27] [47] [46] or on other clinical conditions, such as anemia.[48]

Certainly, addressing food and clean water needs is paramount. Food insecurity increases HIV+ individuals’ vulnerability in developing and developed countries, even in the United States.[49],[50] The deleterious effects of food insecurity include accelerating HIV disease progression at a biological level, weight loss, clinical disease, inability to work and/or riskier activity among sex workers and a cycle of decline leading inexorably to early death[49],[51] and may contribute to non-adherence to ARV.[52],[53] Wasting and cachexia still constitute a significant source of mortality, often in settings of adequate caloric intake.[54]

### Strengths

Our findings are robust to the statistical approach chosen: both Bayesian and frequentist analyses give similar estimates. Typical for a meta-analysis, our findings are sensitive to assumptions regarding the variability between studies. The effects are significant in spite of our very conservative estimate of the between study-variability in our Bayesian model.

Bayesian evidence synthesis produces a posterior distribution of the probability for each possible value of the summary statistic, in our case the NNT. This gives an intuitive representation of likely effect sizes. Bayesian credible intervals provide evidence in favor of the null hypothesis instead of just a rejection of the null hypothesis. To reflect this difference we present the Bayesian estimates with posterior density strips.[55] To aid the reader we have also marked the 95% equal-tailed Bayesian credible interval on each density strip (Figs. 2 and 3). By definition they contain the true value with a 95% probability, giving a clearer indication of the likely range of results in our meta-analysis.[56],[57]

### Limitations

The included studies were conducted in different settings (industrialized versus developing countries). On one hand, the populations studied in the included trials differ, not only in gender but also in racial, ethnic or genetic factors and, to some degree, nutritional status. On the other hand, the baseline BMI in the gay men in California was around 24 versus 23 kg/m^2^ among the Tanzanian women, suggesting similar nutritional status, as other data support.[58],[59]

The MNS interventions varied in content and strength. Some included minerals such as iron, zinc and selenium while others did not. However, for the progression analysis, there were greater similarities in the intervention used, including in terms of dosage.

Our conclusions are somewhat weakened by the small number of included studies; meta-analysis of only few studies may exaggerate the effect estimate.[60] The inclusion of an observational study may be controversial, however, arguments support such inclusion with appropriate precautions.[61],[62],[63] What is more, omission of the one observational study only increased the estimate of the effect of MVM in HIV. While we did not detect significant evidence for heterogeneity in the effect size for progression between the studies, lack of evidence for heterogeneity is not proof of homogeneity. However, the consistency of the treatment effect across different populations and interventions supports the robustness of our findings and their generalizability across different settings.[64]

## Conclusions

We recommend micronutrient supplementation among HIV+ adults not on ARV therapy. Some 35 million people are living with HIV, millions of whom despite clinical eligibility, are yet to be treated. While food and clean water as well as access to ARV remain priorities, potentially cost-effective micronutrient supplementation to slow disease progression and reduce mortality is compelling. Further studies of optimal micronutrient formulae among individuals receiving ARV are warranted.

## Supporting Information

S1 Fig Forest plot of rate of HIV disease progression, frequentist analysis. A frequentist analysis yielded a similar 40% reduction in the rate of progression to clinical disease stages (RR 0.60, 95% CI 0.46, 0.78; p = 0.00008) for subjects on MNS, when including supplement arms that included a MNS alone or MNS plus either zinc or selenium.
(TIFF)

S2 Fig Forest plot of mortality, frequentist analysis. Frequentist analysis of mortality across studies yielded a RR of 0.87 (0.73, 1.04; p = 0.12).
(TIFF)

S3 Fig Frequentist Analysis Controlling for Baseline Co-infection—Without TB. A study level sensitivity analysis excluding studies that included TB and HIV co-infected patients at baseline under frequentist analysis yielded a RR of mortality of 0.70 (0.53, 0.93; p = 0.02).
(EPS)

S4 Fig Frequentist Analysis Controlling for Baseline Co-infection—With TB. In studies with patients with TB at baseline, the impact of MNS on mortality was a RR of 0.91 (0.64, 1.31, p = 0.62).
(EPS)

S1 PRISMA Checklist PRISMA checklist. (DOC)

S1 Table Newcastle-Ottawa Scale for Cohort Studies. (DOCX)

## References

[1] Farmer PE (2013) Chronic Infectious Disease and the Future of Health Care Delivery. N Engl J Med 369: 2424–2436. 10.1056/NEJMsa1310472 24350951

[2] UNAIDS (2013) Global Report: UNAIDS Report on the Global AIDS Epidemic: 2013.

[3] World Health Organization (2014) Global Update on the health sector response to HIV. Available: http://apps.who.int/iris/bitstream/10665/128494/1/9789241507585_eng.pdf?ua=1. Accessed 2015 Jan 6.

[4] World Health Organization (2013) Global Summary of the AIDS Epidemic: 2013. Available: http://www.who.int/hiv/data/epi_core_dec2014.png?ua=1. Accessed 2014 Dec 5).

[5] Nunnari G, Coco C, Pinzone MR, Pavone P, Berretta M, Di Rosa M, et al (2012) The role of micronutrients in the diet of HIV-1-infected individuals. Front Biosci (Elite Ed) 4: 2442–2456. 22652651 10.2741/e556

[6] Singhal N, Austin J (2002) A clinical review of micronutrients in HIV infection. J Int Assoc Physicians AIDS Care (Chic) 1: 63–75. 12942678 10.1177/154510970200100205

[7] [No author listed.] (2004) Complications & side effects. Supplements and CD4+ counts: study finds surprising results. TreatmentUpdate 16: 4–6. 17219668

[8] Lorenc A RN (2013) A review of the use of complementary and alternative medicine and HIV: issues for patient care. AIDS Patient Care STDS 27: 503–510. 10.1089/apc.2013.0175 23991688 PMC3760022

[9] Irlam JH, Visser MM, Rollins NN, Siegfried N (2010) Micronutrient supplementation in children and adults with HIV infection. Cochrane Database Syst Rev: CD003650 10.1002/14651858.CD003650.pub3 21154354

[10] Sutton AJ, Abrams KR (2001) Bayesian methods in meta-analysis and evidence synthesis. Stat Methods Med Res 10: 277–303. 11491414 10.1177/096228020101000404

[11] Baum MK, Campa A, Lai S, Sales Martinez S, Tsalaile L, Burns P, et al (2013 11 27) Effect of micronutrient supplementation on disease progression in asymptomatic, antiretroviral-naive, HIV-infected adults in Botswana: a randomized clinical trial. JAMA 310: 2154–2163. 10.1001/jama.2013.280923 24281460 PMC4347896

[12] Carter G, Sacks H, Indyk D, Suslov K, Johnson M, Andreae M. (2011) Micronutrient supplementation in HIV disease PROSPERO International Prospective Register of Systematic Reviews. PROSPERO 2011:CRD42011001522 PROSPERO 2011:CRD42011001522

[13] Moher D, Liberati A, Tetzlaff J, Altman DG; PRISMA Group. (2009) Preferred reporting items for systematic reviews and meta-analyses: the PRISMA statement. PLoS Med 6. PMC3090117 21603045

[14] Jadad AR, Moore RA, Carroll D, Jenkinson C, Reynolds DJ, Gavaghan DJ, et al (1996) Assessing the quality of reports of randomized clinical trials: is blinding necessary? Control Clin Trials 17: 1–12. 8721797 10.1016/0197-2456(95)00134-4

[15] Stroup DF, Berlin JA, Morton SC, Olkin I, Williamson GD, Rennie D, et al (2000) Meta-analysis of observational studies in epidemiology: a proposal for reporting. Meta-analysis Of Observational Studies in Epidemiology (MOOSE) group. JAMA 283: 2008–2012. 10789670 10.1001/jama.283.15.2008

[16] Deeks JJ, Dinnes J, D'Amico R, Sowden AJ, Sakarovitch C, Song F, et al (2003) Evaluating non-randomised intervention studies. Health Technol Assess 7. 10.3310/hta7270 14499048

[17] Lunn D, Spiegelhalter D, Thomas A and Best N (2009) The BUGS project: Evolution, critique and future directions (with discussion) Statistics in Medicine 28: 3049–3082. 10.1002/sim.3680 19630097

[18] Fawzi WW, Msamanga GI, Spiegelman D, Wei R, Kapiga S, Villamor E, et al (2004) A randomized trial of multivitamin supplements and HIV disease progression and mortality. N Engl J Med 351: 23–32. 15229304 10.1056/NEJMoa040541

[19] Kass R, Raftery A (1995) Bayes Factors. Journal of the American Statistical Association 90: 773–795.

[20] Egger M, Davey-Smith G, Schneider M, Minder C (1997) Bias in meta-analysis detected by a simple, graphical test. BMJ 315: 629–634. 9310563 10.1136/bmj.315.7109.629 PMC2127453

[21] Abrams B, Duncan D, Hertz-Picciotto I (1993) A prospective study of dietary intake and acquired immune deficiency syndrome in HIV-seropositive homosexual men. J Acquir Immune Defic Syndr 6: 949–958. 8100273

[22] Baum MK, Campo A, Lai S, Sales S, Li Y, Burns P, et al and the Dikotlana Study Team (2010) Micronutrient supplementation to prevent disease progression in HIV infected adults in Botswana XVII International AIDS Conference. Vienna, Austria: International AIDS Society.

[23] Jiamton S, Pepin J, Suttent R, Filteau S, Mahakkanukrauh B, Hanshaoworakul W et al (2003) A randomized trial of the impact of multiple micronutrient supplementation on mortality among HIV-infected individuals living in Bangkok. AIDS 17: 2461–2469. 14600517 10.1097/00002030-200311210-00008

[24] Kelly P, Katubulushi M, Todd J, Banda R, Yambayamba V, Fwoloshi M, et al (2008) Micronutrient supplementation has limited effects on intestinal infectious disease and mortality in a Zambian population of mixed HIV status: a cluster randomized trial. Am J Clin Nutr 88: 1010–1017. 18842788 10.1093/ajcn/88.4.1010 PMC2777266

[25] Range N, Changalucha J, Krarup H, Magnussen P, Andersen AB, Friis H. (2006) The effect of multi-vitamin/mineral supplementation on mortality during treatment of pulmonary tuberculosis: a randomised two-by-two factorial trial in Mwanza, Tanzania. Br J Nutr 95: 762–770. 16571156 10.1079/bjn20051684

[26] Semba RD, Ricketts EP, Mehta S, Netski D, Thomas D, Kirk G, et al (2007) Effect of micronutrients and iron supplementation on hemoglobin, iron status, and plasma hepatitis C and HIV RNA levels in female injection drug users: a controlled clinical trial. J Acquir Immune Defic Syndr 45: 298–303. 17414930 10.1097/QAI.0b013e318050d698

[27] Villamor E, Mugusi F, Urassa W, Bosch RJ, Saathoff E, Matsumoto K, et al (2008) A trial of the effect of micronutrient supplementation on treatment outcome, T cell counts, morbidity, and mortality in adults with pulmonary tuberculosis. J Infect Dis 197: 1499–1505. 10.1086/587846 18471061 PMC2564793

[28] Shekelle PG, Morton SC, Suttorp MJ, Buscemi N, Friesen C (2005) Challenges in systematic reviews of complementary and alternative medicine topics. Ann Intern Med 142: 1042–1047. 15968028 10.7326/0003-4819-142-12_part_2-200506211-00003

[29] Carter G, Suslov K, Johnson M, Andreae M, Sacks H. (2012) Multiple Micronutrients in HIV Disease: A Bayesian Meta-Analysis In: Society IA, editor. XIX International AIDS Conference. Washington, DC: International AIDS Society pp. THPE053.

[30] Semba RD, Kumwenda J, Zijlstra E, Ricks MO, van Lettow M, Whalen C, et al (2007) Micronutrient supplements and mortality of HIV-infected adults with pulmonary TB: a controlled clinical trial. Int J Tuberc Lung Dis 11: 854–859. 17705950

[31] Fawzi WW, Msamanga GI, Hunter D, Renjifo B, Antelman G, Bang H, et al (2002) Randomized trial of vitamin supplements in relation to transmission of HIV-1 through breastfeeding and early child mortality. AIDS 16: 1935–1944. 12351954 10.1097/00002030-200209270-00011

[32] McClelland RS, Baeten JM, Overbaugh J, Richardson BA, Mandaliya K, Emery S, et al (2004) Micronutrient supplementation increases genital tract shedding of HIV-1 in women: results of a randomized trial. J Acquir Immune Defic Syndr 37: 1657–1663. 15577425 10.1097/00126334-200412150-00021

[33] Kapp C (2005) SA health minister urged to stop vitamin-peddling doctor. Lancet 366: 1837–1838. 16315355 10.1016/S0140-6736(05)67739-2

[34] Marston B, De Cock KM (2004) Multivitamins, nutrition, and antiretroviral therapy for HIV disease in Africa. N Engl J Med 351: 78–80. 15229312 10.1056/NEJMe048134

[35] Jiamton S, Chaisilwattana P, Pepin J, Suttent R, Mahakkanukrauh B, Filteau S, et al (2004) A randomized placebo-controlled trial of the impact of multiple micronutrient supplementation on HIV-1 genital shedding among Thai subjects. J Acquir Immune Defic Syndr 37: 1216–1218. 15319683 10.1097/01.qai.0000136092.79153.ac

[36] Bhutta ZA, Das JK, Rizvi A, Gaffey MF, Walker N, Horton S, et al (2013) Evidence-based interventions for improvement of maternal and child nutrition: what can be done and at what cost? Lancet 382: 452–477. 10.1016/S0140-6736(13)60996-4 23746776

[37] Asdamongkol N, Phanachet P, Sungkanuparph S. (2013) Low Plasma Zinc Levels and Immunological Responses to Zinc Supplementation in HIV-Infected Patients with Immunological Discordance after Antiretroviral Therapy. Jpn J Infect Dis 66: 469–474. 24270132 10.7883/yoken.66.469

[38] Childs K, Welz T, Samarawickrama A, Post FA (2012) Effects of vitamin D deficiency and combination antiretroviral therapy on bone in HIV-positive patients. AIDS 26: 253–262. 10.1097/QAD.0b013e32834f324b 22112601

[39] Havens PL, Mulligan K, Hazra R, Flynn P, Rutledge B, Van Loan MD, et al (2012) Serum 25-hydroxyvitamin D response to vitamin D3 supplementation 50,000 IU monthly in youth with HIV-1 infection. J Clin Endocrinol Metab 97: 4004–4013. 10.1210/jc.2012-2600 22933542 PMC3485594

[40] Fawzi W, Msamanga G, Antelman G, Xu C, Hertzmark E, Spiegelman D, et al (2004) Effect of prenatal vitamin supplementation on lower-genital levels of HIV type 1 and interleukin type 1 beta at 36 weeks of gestation. Clin Infect Dis 38: 716–722. 14986257 10.1086/381673

[41] McGrath N, Bellinger D, Robins J, Msamanga GI, Tronick E, Fawzi W. (2006) Effect of maternal multivitamin supplementation on the mental and psychomotor development of children who are born to HIV-1-infected mothers in Tanzania. Pediatrics 117: e216–225. 16452331 10.1542/peds.2004-1668

[42] Olofin IO, Spiegelman D, Aboud S, Duggan C, Danaei G, Fawzi WW. (2014) Supplementation With Multivitamins and Vitamin A and Incidence of Malaria Among HIV-Infected Tanzanian Women. J Acquir Immune Defic Syndr 67: S173–S178. 10.1097/QAI.0000000000000375 25436815 PMC4251912

[43] Kaiser JD, Campa AM, Ondercin JP, Leoung GS, Pless RF, Baum MK. (2006) Micronutrient supplementation increases CD4 count in HIV-infected individuals on highly active antiretroviral therapy: a prospective, double-blinded, placebo-controlled trial. J Acquir Immune Defic Syndr 42: 523–528. 16868496 10.1097/01.qai.0000230529.25083.42

[44] Doitsh G, Galloway N, Geng X, Yang Z, Monroe KM, Zepeda O, et al (2013) Cell death by pyroptosis drives CD4 T-cell depletion in HIV-1 infection. Nature. 10.1038/nature12940 PMC4047036 24356306

[45] Cunningham-Rundles S, McNeeley DF, Moon A (2005) Mechanisms of nutrient modulation of the immune response. J Allergy Clin Immunol 115: 1119–1128; quiz 1129. 15940121 10.1016/j.jaci.2005.04.036

[46] Chilenje Infant Growth, Nutrition and Infection (CIGNIS) Study Team (2010) Micronutrient fortification to improve growth and health of maternally HIV-unexposed and exposed Zambian infants: a randomised controlled trial. PLoS One 5: e11165 10.1371/journal.pone.0011165 20567511 PMC2887362

[47] Isanaka S, Mugusi F, Hawkins C, Spiegelman D, Okuma J, Aboud S, et al (2012) Effect of high-dose vs standard-dose multivitamin supplementation at the initiation of HAART on HIV disease progression and mortality in Tanzania: a randomized controlled trial. JAMA 308: 1535–1544. 10.1001/jama.2012.13083 23073950 PMC3811009

[48] Fawzi WW, Msamanga GI, Kupka R, Spiegelman D, Villamor E, Mugusi F, et al (2007) Multivitamin supplementation improves hematologic status in HIV-infected women and their children in Tanzania. Am J Clin Nutr 85: 1335–1343. 17490971 10.1093/ajcn/85.5.1335

[49] Weiser SD, Hatcher A, Frongillo EA, Guzman D, Riley ED, Bangsberg DR, et al (2013) Food insecurity is associated with greater acute care utilization among HIV-infected homeless and marginally housed individuals in San Francisco. J Gen Intern Med 28: 91–98. 10.1007/s11606-012-2176-4 22903407 PMC3539018

[50] Tiyou A, Belachew T, Alemseged F, Biadgilign S (2012) Food insecurity and associated factors among HIV-infected individuals receiving highly active antiretroviral therapy in Jimma zone Southwest Ethiopia. Nutr J 11: 51 10.1186/1475-2891-11-51 22824145 PMC3507894

[51] Shannon K, Kerr T, Milloy MJ, Anema A, Zhang R, Montaner JS, et al (2011) Severe food insecurity is associated with elevated unprotected sex among HIV-seropositive injection drug users independent of HAART use. AIDS 25: 2037–2042. 10.1097/QAD.0b013e32834b35c9 21811140 PMC3956106

[52] de Pee S, Grede N, Mehra D, Bloem MW. (2014) The Enabling Effect of Food Assistance in Improving Adherence and/or Treatment Completion for Antiretroviral Therapy and Tuberculosis Treatment: A Literature Review. AIDS Behav Epub ahead of print]. 10.1007/s10461-014-0730-2 24619602

[53] Berhe N, Tegabu D, Alemayehu M (2013) Effect of nutritional factors on adherence to antiretroviral therapy among HIV-infected adults: a case control study in Northern Ethiopia. BMC Infect Dis 13: 233 10.1186/1471-2334-13-233 23701864 PMC3669031

[54] Kouanda S, Meda IB, Nikiema L, Tiendrebeogo S, Doulougou B, Kabore I, et al (2012) Determinants and causes of mortality in HIV-infected patients receiving antiretroviral therapy in Burkina Faso: a five-year retrospective cohort study. AIDS Care 24: 478–490. 10.1080/09540121.2011.630353 22148973

[55] Jackson CH (2008) Displaying Uncertainty With Shading. The American Statistician 62: 340–347.

[56] Kruschke JK. (2010) Doing Bayesian Data Analysis: A Tutorial with R and BUGS. Cambridge, MA: Elsevier.

[57] Wagenmakers E-J, Lee M. D., Lodewyckx T., & Iverson G. (2008) Bayesian versus frequentist inference In Hoijtink H., Klugkist I., and Boelen P. A. (Eds.), Bayesian Evaluation of Informative Hypotheses, pp. 181–207. Springer: New York.

[58] Keding GB, Msuya J, Maass BL, Krawinkel MB. (2011) Dietary patterns and nutritional health of women: the nutrition transition in rural Tanzania. Food Nutr Bull 32: 218–226. 22073796 10.1177/156482651103200306

[59] Liu E, Spiegelman D, Semu H, Hawkins C, Chalamilla G, Aveika A, et al (2011) Nutritional status and mortality among HIV-infected patients receiving antiretroviral therapy in Tanzania. J Infect Dis 204: 282–290. 10.1093/infdis/jir246 21673040

[60] Myers EF, Parrott JS, Cummins DS, Splett P (2011) Funding source and research report quality in nutrition practice-related research. PLoS One 6: e28437 10.1371/journal.pone.0028437 22163017 PMC3232225

[61] Shrier I, Boivin J, Steele RJ, Platt RW, Furlan A, Kakuma R, et al (2007) Should meta-analyses of interventions include observational studies in addition to randomized controlled trials? A critical examination of underlying principles. Am J Epidemiol 166: 1203–1209. 17712019 10.1093/aje/kwm189

[62] Peinemann F, Tushabe D, Kleijnen J. (2013) Using multiple types of studies in systematic reviews of health care interventions—a systematic review. PLoS One 8: e85035 10.1371/journal.pone.0085035 24416098 PMC3887134

[63] Ioannidis JP, Patsopoulos N, Rothstein HR. (2008) Reasons or excuses for avoiding meta-analysis in forest plots. British Medical Journal 336: 1413–1415. 10.1136/bmj.a117 18566080 PMC2432114

[64] Kish L (1987, 2004) Statistical Design for Research; Chapter 1, Representation, Randomization and Realism. New York: John Wiley & Sons, Inc.

