# Correction: Micronutrients in HIV: A Bayesian Meta-Analysis

**DOI:** 10.1371/journal.pone.0148392

**Published:** 2016-01-28

**Authors:** George M. Carter, Debbie Indyk, Matthew Johnson, Michael Andreae, Kathryn Suslov, Sudharani Busani, Aryan Esmaeili, Henry S. Sacks

The density strip in Fig 2 is incorrect. Please view the correct Fig 2 here.

**Fig 2:**
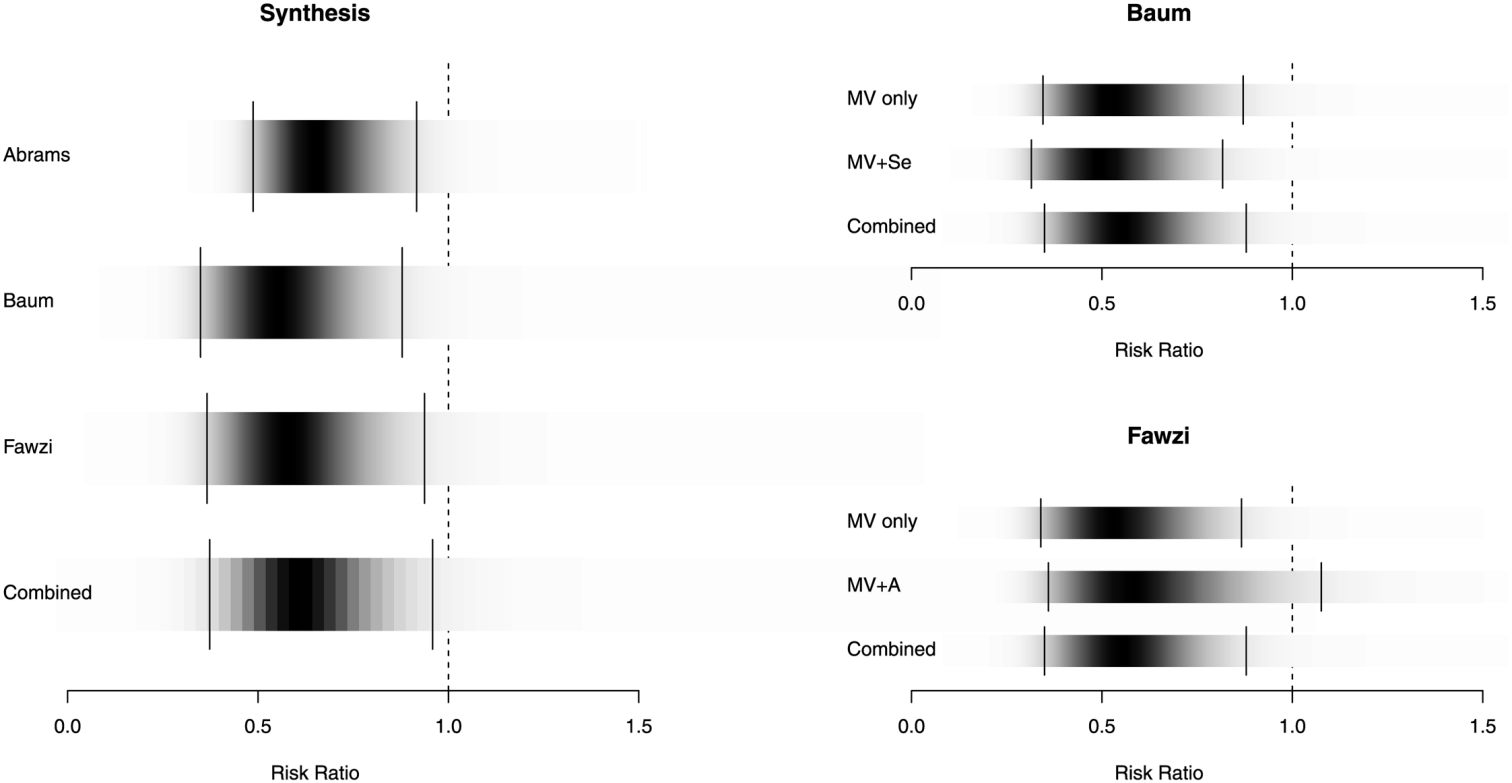
Density Strip Plot of Relative Risk of Progression to Clinical Disease/AIDS. Bayesian random effects analysis (favors treatment on the left of 1.0); density plots on the figure on the left represent combined data; on the right accounts for individual study arms and the impact of adding selenium or vitamin A to MNS [55].
