# Supplementary figures and images for: Micronutrients in HIV: A Bayesian Meta-Analysis
Source: PLoS One. 2015 Apr 1;10(4):e0120113. doi: 10.1371/journal.pone.0120113 (PMC4382132; doi:10.1371/journal.pone.0120113)

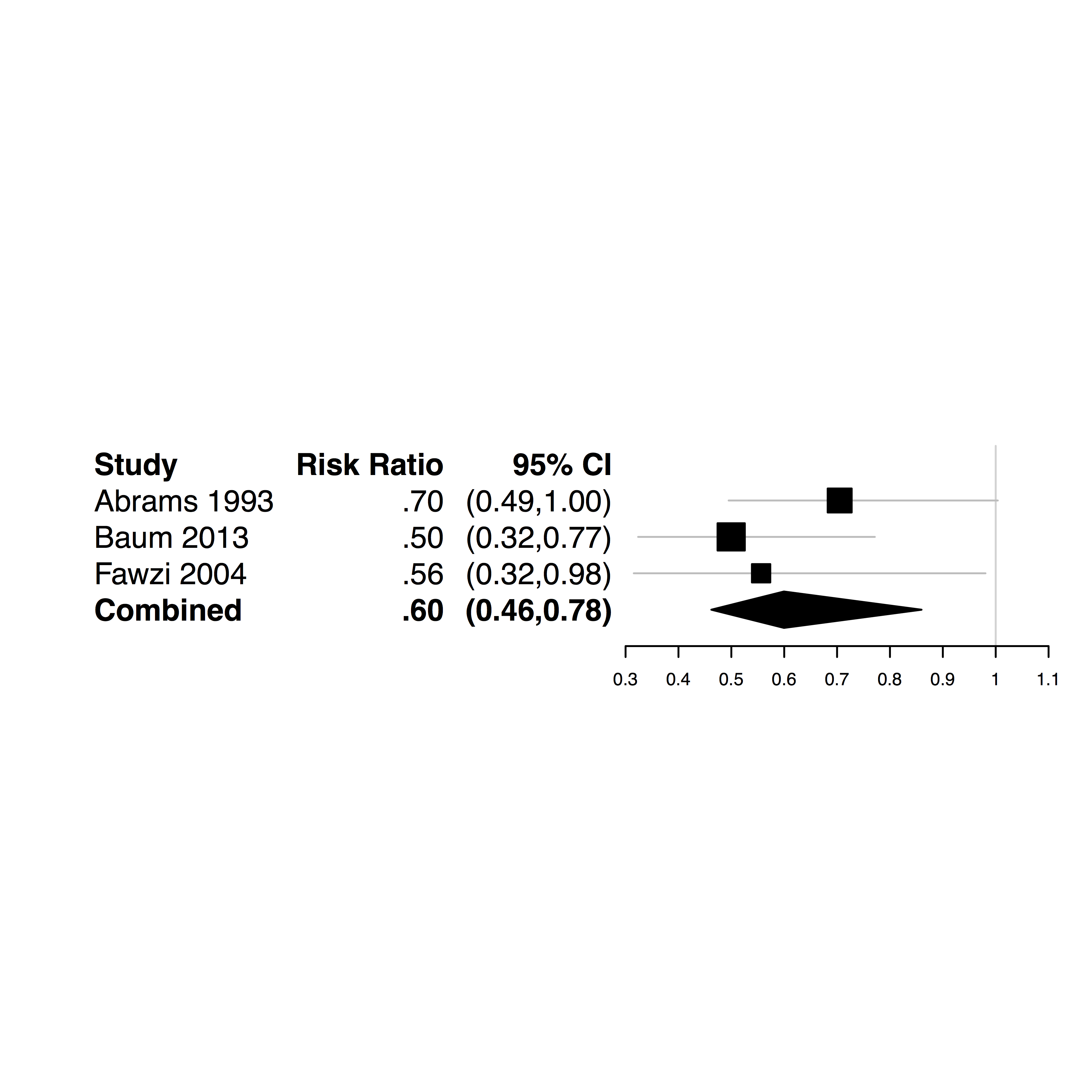

Supplement: S1 Fig — A frequentist analysis yielded a similar 40% reduction in the rate of progression to clinical disease stages (RR 0.60, 95% CI 0.46, 0.78; p = 0.00008) for subjects on MNS, when including supplement arms that included a MNS alone or MNS plus either zinc or selenium. (TIFF) [file pone.0120113.s001.tiff]

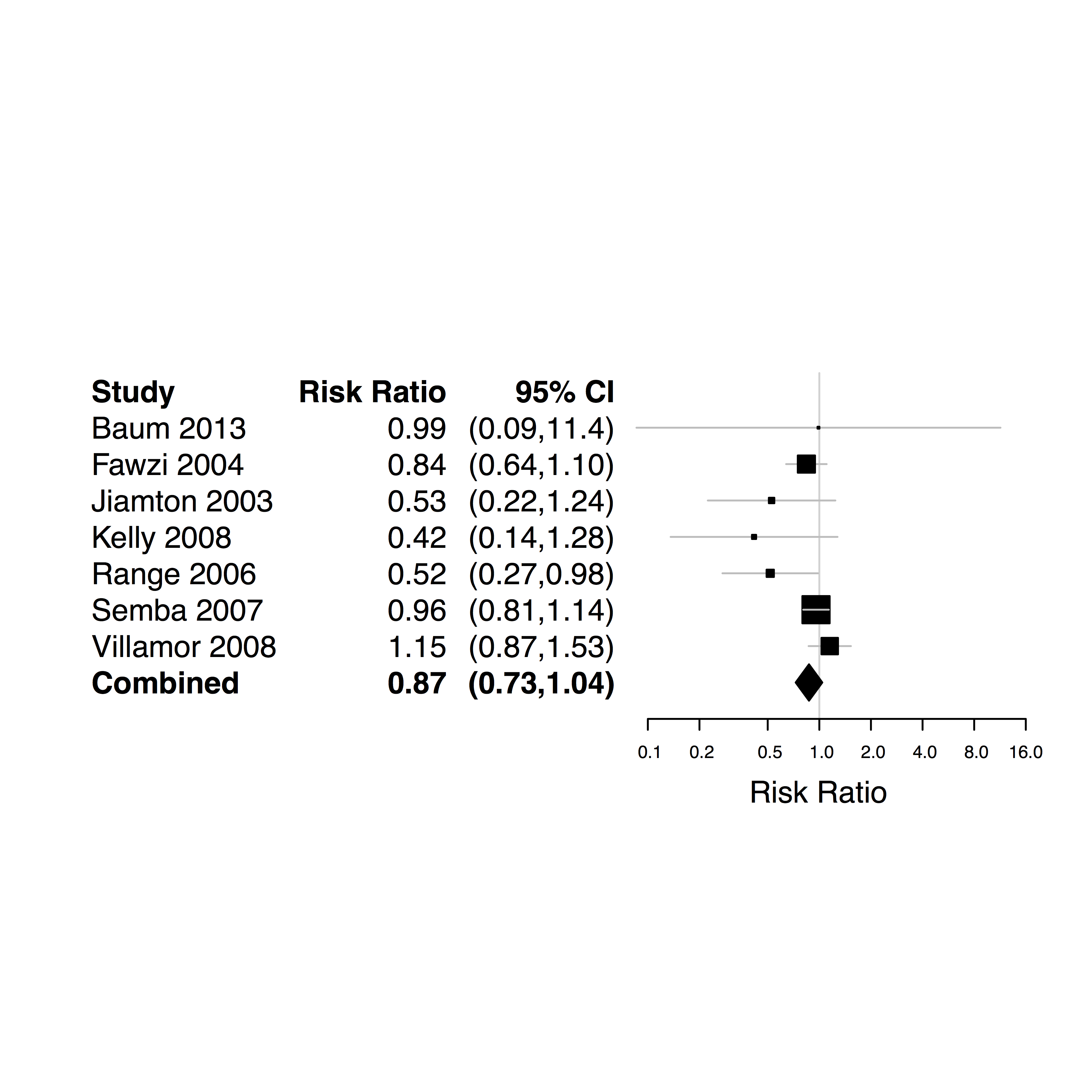

Supplement: S2 Fig — Frequentist analysis of mortality across studies yielded a RR of 0.87 (0.73, 1.04; p = 0.12). (TIFF) [file pone.0120113.s002.tiff]
