# Supplementary material for: Micronutrients in HIV: A Bayesian Meta-Analysis
Source: PLoS One. 2015 Apr 1;10(4):e0120113. doi: 10.1371/journal.pone.0120113 (PMC4382132; doi:10.1371/journal.pone.0120113)
Supplement: S1 Table — (DOCX) [file pone.0120113.s006.docx]

**S1 Table**

**Newcastle-Ottawa Scale for Cohort Studies**

| **Criterion** | **Y/N** | **Rationale** |
| --- | --- | --- |
| ***Selection*** |  |  |
| 1) Representativeness of the Exposed Cohort | N | Adults with HIV not on ARV |
| 2) Selection of the Non-Exposed Cohort | N | Randomly by self-report |
| 3) Ascertainment of Exposure | N | Modified version of the nutrition portion of the Health Habits and History Questionnaire |
| 4) Demonstration That Outcome of Interest Was Not Present at Start of Study | Y | Yes; people with low CD4, clinical AIDS excluded |
| ***Comparability*** |  |  |
| 1) Comparability of Cohorts on the Basis of the Design or Analysis | Y,Y | Adjusted for CD4, age, other factors; however: "*Other characteristics, including … use of zidovudine were also examined but these variables were not included as confounders in the final models because their inclusion in preliminary models did not alter the findings of any of the nutrients."* |
| ***Outcome*** |  |  |
| 1) Assessment of Outcome | Y | Well described methodology for assessing progression |
| 2) Was Follow-Up Long Enough for Outcomes to Occur | Y | 6 years |
| 3) Adequacy of Follow Up of Cohorts | N | Follow-ups accounted for |
| **TOTAL** | **5** |  |
